# Emerging Molecular Dependencies of Mutant EGFR-Driven Non-Small Cell Lung Cancer

**DOI:** 10.3390/cells10123553

**Published:** 2021-12-16

**Authors:** Dylan A. Farnsworth, Yankuan T. Chen, Georgia de Rappard Yuswack, William W. Lockwood

**Affiliations:** 1 Department of Integrative Oncology, BC Cancer Research Institute, Vancouver, BC V5Z 1L3, Canada; terrych1@student.ubc.ca (Y.T.C.); gderappard@bccrc.ca (G.d.R.Y.); 2 Department of Pathology & Laboratory Medicine, University of British Columbia, Vancouver, BC V6T 1Z7, Canada

**Keywords:** EGFR, TKI, lung cancer, functional genomics, CRISPR screens

## Abstract

Epidermal growth factor receptor (EGFR) mutations are the molecular driver of a subset of non-small cell lung cancers (NSCLC); tumors that harbor these mutations are often dependent on sustained oncogene signaling for survival, a concept known as “oncogene addiction”. Inhibiting EGFR with tyrosine kinase inhibitors has improved clinical outcomes for patients; however, successive generations of inhibitors have failed to prevent the eventual emergence of resistance to targeted agents. Although these tumors have a well-established dependency on EGFR signaling, there remain questions about the underlying genetic mechanisms necessary for EGFR-driven oncogenesis and the factors that allow tumor cells to escape EGFR dependence. In this review, we highlight the latest findings on mutant EGFR dependencies, co-operative drivers, and molecular mechanisms that underlie sensitivity to EGFR inhibitors. Additionally, we offer perspective on how these discoveries may inform novel combination therapies tailored to EGFR mutant NSCLC.

## 1. Introduction

Lung cancer remains the leading cause of cancer-related deaths worldwide [1]. In non-small cell lung cancer (NSCLC) patients, activating mutations in the epidermal growth factor receptor (EGFR) were found to be associated with response to EGFR inhibition [2]. This molecular dependency has led to EGFR mutant tumors being considered “oncogene addicted” and has informed therapeutic approaches based on targeting driver mutations with small molecule inhibitors. In EGFR mutant NSCLC, EGFR tyrosine kinase inhibitors (TKIs) have greatly improved patient outcomes relative to standard chemotherapy [3,4,5]. However, there remain questions about the molecular factors that regulate the dependency of tumors on mutant EGFR. One underlying assumption of targeted therapy is that EGFR mutant tumors are solely reliant on EGFR for survival. While this may be true for most patients, not all respond to EGFR inhibition in a primary setting. Osimertinib is a third-generation EGFR TKI and the current standard of care for EGFR mutant tumors, and it was found to have an 80% objective response rate (ORR) in a phase-3 trial of previously untreated EGFR mutant NSCLC [5], suggesting intrinsic mechanisms of resistance to EGFR inhibition. In addition, patients who do initially respond to EGFR inhibition inevitably develop adaptive resistance by various mechanisms that reactivate or bypass EGFR signaling [6]. Finally, while it is established that EGFR is a driver oncogene and has a key molecular dependency in EGFR mutant tumors, research has shown that EGFR mutation alone is not sufficient to transform normal cells [7,8,9]. Together, this highlights the role of additional genetic and molecular modifications in mediating both EGFR mutant tumor progression and in allowing cells to bypass or reactivate EGFR signaling in the context of targeted therapy.

In this review, we highlight the most current research on molecular factors that modulate sensitivity to EGFR inhibition and cellular dependence on mutant EGFR signaling and the co-operative genetic drivers necessary for EGFR-driven tumorigenesis. Additionally, we offer insight into how these discoveries may translate to therapeutic strategies by combination-based targeting of both EGFR and its associated dependencies.

## 2. EGFR Driver Mutations in NSCLC

**Figure 1:**
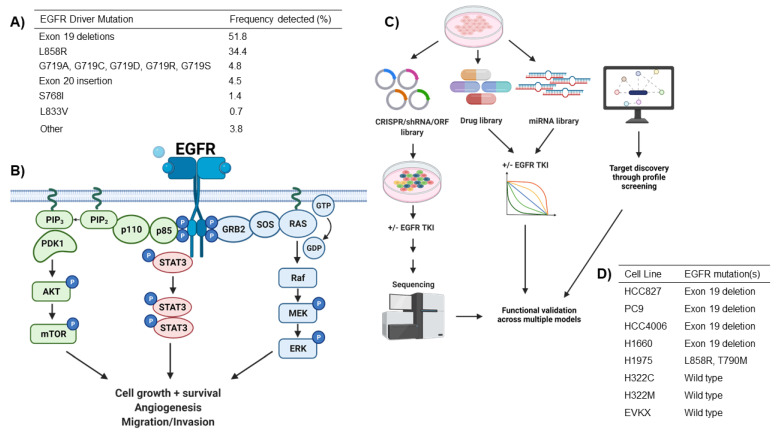
Methodology used to investigate factors that affect mutant EGFR dependency in NSCLC. (**A**) Commonly detected EGFR mutations in NSCLC patients. (**B**) Mutant EGFR drives signaling through the RAS/MAPK pathway, the PI3K/AKT pathway, and the STAT3 pathway. The combined result is a promotion of cell proliferation, cell survival, angiogenesis, and increased migration and invasion. (**C**) High throughput screening techniques used to identify dependencies of EGFR mutant NSCLC. EGFR mutant cells are screened by transducing them with lentivirus containing CRISPR guides, shRNAs, and/or ORFs of varying coverage across the genome. The cells are then treated with EGFR TKIs, left to proliferate, then harvested and sequenced, to which guides/shRNAs/ORFs are enriched or depleted in the population of cells that survives the selective pressure of EGFR TKIs. Determinants of EGFR dependency are also screened for by treating cells with a library of drugs or miRNA libraries in combination with EGFR TKIs and examining which drugs/miRNAs synergize with or antagonize EGFR inhibitors. EGFR dependencies are also investigated by using bioinformatics profiling approaches. Using sequencing data, gene protein interaction data, and drug-protein interaction maps, researchers can determine proteins that are associated with drug-resistant phenotypes and which drugs target those genes. (**D**) Cell lines used for the study of EGFR dependencies and their EGFR mutation status. Figure made with BioRender.

Somatic mutations in EGFR occur in around 15–30% of lung adenocarcinomas (LUADs), the major subtype of lung cancer. Most mutations detected in patients are found in exons 18 through 21, which encode the tyrosine kinase domain of EGFR [10]. The two most common and best-characterized mutations are in-frame deletions in exon 19 (ex19del), followed by the L858R missense mutation. G718X substitutions and other insertion, deletions, and missense mutations in exons 19 through 21 are also detected but less commonly [10] (Figure 1A). These activating mutations all affect key structures involving the ATP binding cleft of the tyrosine kinase domain [11]. Tyrosine kinase inhibitors have proven to be effective in the treatment of EGFR mutant NSCLCs as these mutations render the protein constitutively active, regardless of ligand binding or dimerization [12]. Mutant EGFR subsequently transduces signals through multiple pathways, including the MAPK/ERK/AKT and STAT3 nodes (Figure 1B) [13,14,15]. The overall result is the promotion of cell growth and survival, alongside angiogenesis, tumor migration, and invasion [16].

First-generation TKIs, which include gefitinib and erlotinib, target the ATP binding site of EGFR by competitive, reversible inhibition [17,18]. Second-generation inhibitors such as afatinib irreversibly bind EGFR at the cysteine residue at C797 within the kinase domain. Although initial objective response rates are strong with both generations of inhibitors, resistance through the gatekeeper T790M mutation occurs in around 60%, along with other mechanisms of resistance [19]. Osimertinib, a third-generation EGFR inhibitor, can re-sensitize cells bearing the T790M mutation to EGFR inhibition [20] and has been demonstrated to have an ORR of 71% in patients bearing the T790M mutation who progressed after treatment with earlier generation TKIs [21]. Although it is now given as first-line therapy for EGFR mutant NSCLC [5], patients inevitably develop resistance through single amino acid substitutions at the EGFR cysteine residue C797 as well as other mechanisms that bypass dependence of the cancer cells on EGFR signaling for survival [6].

Mechanisms of resistance to EGFR therapies can be challenging to decipher in a clinical setting. In around 40–50% of patients whose tumors are sequenced at relapse to TKI treatment, no known mechanisms of resistance are detected [6]. Among a population of EGFR mutant NSCLC patients, a broad range of amplification, mutations, and other genetic changes has been detected following progression after treatment with EGFR TKIs [6]. Characterized resistance mechanisms to osimertinib, the current standard of care, include amplifications of cell cycle genes CCND1 and CDK4 [22,23]; amplification of the receptor tyrosine kinases MET [23,24,25] and HER2 [23]; acquired oncogenic fusions in ALK [26], RET [24,27], ROS1 [28], and NTRK1 [23]; mutations in BRAF [23], KRAS [23,25], and PI3K [23,24,25]; loss of PTEN [29]; acquired secondary/tertiary EGFR mutations [23,24,25]; and phenotypic transformation to small cell lung cancer [30,31], all of which have been validated as acquired resistance mechanisms in pre-clinical models. Intrinsic resistance also limits the efficacy of EGFR inhibition. In the phase-3 FLAURA trial, first-line osimertinib in EGFR mutant NSCLC patients has an ORR of 80%, indicating that a portion of patients do not respond to initial treatment [5]. In addition to non-responders, a subset of patients experience early relapse within 6 months [32]. Studies have identified factors that mediate intrinsic resistance to EGFR TKIs in some of these instances. A meta-analysis performed on clinical trial data revealed that a *BIM* deletion polymorphism is associated with shorter progression free survival and overall survival in EGFR mutant NSCLC patients treated with TKIs [33]. Additionally, the receptor tyrosine kinase AXL was identified as mediating intrinsic resistance to osimertinib in pre-clinical models by driving EGFR and HER3 signaling [34]. MET amplification has been reported in patients as an acquired genetic alteration following EGFR TKI treatment; however MET signaling was also found to confer intrinsic resistance to EGFR inhibition, suggesting a context-dependent role in EGFR TKI resistance [35].

While some of these genetic alterations yield sensitivity to alternative targeted therapies, successfully detecting these mutations, personalizing treatments, and validating dosage for all different drug combinations to combat resistance mechanisms while also preventing reactivation of EGFR signaling remains challenging. Furthermore, EGFR mutant tumors respond poorly to immune checkpoint inhibitors [36,37,38,39]; this is attributed to factors such as lower tumor mutation burdens in EGFR mutant lung cancer [40], but this remains poorly understood. As such, chemotherapy is the only approved treatment option for patients who progress on from EGFR TKIs, leading to poor patient outcomes.

### 2.1. Functional Modifiers of Mutant EGFR-Induced Lung Tumorigenesis as Targets for Therapeutic Intervention

Current efforts to prolong the effect of EGFR treatments are focused on creating more potent TKIs that are insensitive to on-target mutations that drive resistance; however, it is likely that any new inhibitors will also suffer from a new spectrum of uncharacterized resistance mechanisms. Thus, pharmacologically targeting mechanisms that enable intrinsic resistance or tumor cell survival and residual disease after first-line treatment with current inhibitors such as osimertinib may offer a more promising strategy to increase efficacy in patients. While MET and AXL signaling, as well as *BIM* polymorphisms, have been uncovered as mediators of intrinsic resistance, as described above, genetic factors that underly differential sensitivity to EGFR inhibition remain uncharacterized and poorly understood. Advances in library preparation, high-throughput sequencing, and bioinformatics techniques have allowed researchers to perform large-scale unbiased screens using genome-wide CRISPR-Cas9 and shRNA to knockdown genes, cDNA overexpression libraries to express mutated or wild-type genes in cancer cells, and drug libraries to directly inhibit protein functions (Figure 1C,D). In the context of EGFR TKIs, these have been applied to determine which genomic alterations or signaling networks may increase or decrease EGFR dependency and response to TKIs in EGFR mutant NSCLC. Here, we present the top mediators of EGFR-signaling dependency, uncovered from functional genomics studies, highlighting their major implications for future therapeutic strategies (Table 1).

#### 2.1.1. Positive Moderators of EGFR Dependency

##### YAP

In EGFR mutant NSCLC, EGFR TKI-sensitive cells can give way to a population of TKI-resistant drug-tolerant persister (DTP) cells that will proliferate at a low rate when exposed to the drug and become the dominant clonal population after the death of rapidly proliferating EGFR-dependent cells [44]. In the clinic, this may present as a partial objective response or intrinsic resistance. To study this form of resistance, Zeng and colleagues [44] performed a genome-wide CRISPR-Cas9 loss-of-function screen of EGFR mutant HCC827 cells to systematically identify both positive and negative regulators of EGFR dependence. HCC827 cells have a low erlotinib IC_50_; however, when cultured in a clinically relevant concentration (1 µM), approximately 30% of cells can survive the initial pulse of TKI and remain as DTP cells. Cells were transduced with lentivirus containing sgRNAs targeting 18,360 genes, treated with either DMSO or 1 µM erlotinib, and harvested three weeks later to assess the guides enriched or depleted in the DTP cells [44]. Among the guides that were depleted and, as such, predicted to have synergized with erlotinib were *YAP1*, *LPAR2,* and *RIC8A*. LPAR proteins are a family of membrane-bound G-protein-coupled receptors (GPCRs); subsequently, several LPAR antagonists were found to synergize with erlotinib. Among the novel hits, *RIC8A* was found to be synthetic lethal with EGFR inhibition in EGFR mutant NSCLC cells. *RIC8A* functions as a chaperone and GTP exchange factor (GEF) for a subset of GPCRs [58,59] and was found to be a positive regulator of YAP signaling [44]. YAP is one of the main effector proteins of Hippo signaling and translocates to the nucleus to activate transcription of growth-promoting and anti-apoptotic genes [60]. Activation of YAP was previously found to modulate resistance to MEK and BRAF inhibitors in NSCLC [61]. In this study, *RIC8A* was found to positively regulate YAP signaling through the Gα-Rho/Rac axis, and loss of YAP signaling was synthetic lethal, with EGFR inhibition. Interestingly, GPCRs are also known to modulate Rho/RAC GTPase activity, leading to actin cytoskeleton remodeling, which, in turn, regulates YAP [62]. *RIC8A* inhibition also synergized with erlotinib and gefitinib in two other EGFR mutant cell lines, H1975 and H3255. Together, this study suggests a dependence for survival on GPCRs and YAP signaling in EGFR mutant lung cancer.

In a separate screen, Cheng and colleagues [52] also identified *YAP1* as a dependency factor in mutant EGFR addicted cells. This group conducted a screen designed to identify factors that regulate cisplatin sensitivity in NSCLC cells using 60,000 individual shRNAs in PC9 cells, collecting and sequencing the cells 14 days after initial treatment. *YAP1* was identified as a regulator of cisplatin sensitivity [52]; while performing functional validation, YAP1 knockdown by siRNA was also found to further sensitize HCC827 and PC9 cells (both EGFR mutants) to EGFR inhibition with erlotinib. Additionally, immunohistochemistry (IHC) on patient samples revealed positive YAP1 staining in 94% of EGFR mutant cases. Thus, the interplay between EGFR and YAP1 supports a role for YAP1 signaling in EGFR mutant NSCLC. In esophageal cancer, YAP1 was found to mediate EGFR overexpression [63], while a reciprocal relationship was found in hepatocellular carcinoma, where EGFR induced expression of YAP1 [64]. Additionally, cytoplasmic YAP1, the inactive form of the protein, is associated with prolonged survival in LUAD patients treated with TKIs [65]. Overall, this data further supports YAP1 as a positive mediator of EGFR signaling.

##### Wnt/β-Catenin Pathway

In another study aiming to identify genes whose suppression increases the effectiveness of gefitinib, Casás-Selves and colleagues [56] performed a genome-wide shRNA loss-of-function screen in two NSCLC cell lines: H322C and HCC4006. H4006 is an EGFR mutant cell line that is highly sensitive to gefitinib, whereas the H322C cell line is an EGFR^wt^ that still exhibits intermediate sensitivity to gefitinib [56]. Among the depleted shRNAs in the gefinitib-treated populations were shRNAs targeting numerous components of the Wnt/tankyrase/β-catenin pathway, most notably the poly-ADP-ribosylating enzyme TNSK1. The Wnt/β-catenin pathway is a highly conserved signaling transduction cascade that controls myriad cellular functions, including proliferation, survival, migration, and apoptosis [66]. TNSK1 promotes the proteasomal degradation of Axin, in turn destabilizing the β-catenin destruction complex and promoting β-catenin signaling [67]. Inhibition of tankyrase activity by shRNA or with small molecules has minor effects on cell proliferation but, in combination with EGFR inhibition, results in synergistic suppression of cell growth; the inhibition of Wnt or β-catenin was shown to further sensitize cells to EGFR inhibition. Of note, downstream EGFR pathway targets are not affected by the inhibition of the Wnt/tankyrase/β-catenin pathway, suggesting that the effects observed are not from increased EGFR effector inhibition. The anti-proliferative effects can be rescued by expressing an active form of β-catenin, further validating this pathway as a key mediator of EGFR signaling dependence. In vivo, the combination of a *TNSK1*-targeted shRNA and gefitinib results in more substantial tumor growth inhibition than gefitinib alone. These results highlight Wnt/tankyrase/β-catenin as a signaling pathway that allows for NSCLC cells to escape EGFR dependence, serving as a mechanism of resistance to EGFR TKIs. The availability of tankyrase inhibitors makes this a promising therapeutic target if clinical biomarkers for the activation of this pathway in patients are discovered.

##### NF-κB

Bivona et al. [57] utilized a complementary approach to uncover modifiers of mutant EGFR dependence. Instead of examining EGFR mutant NSCLC cells that are sensitive to EGFR inhibition, they utilized H1650 cells, which, despite bearing an in-frame exon 19 deletion mutation in EGFR, are TKI-insensitive. To identify genes that restore EGFR dependence when silenced, they introduced a pooled shRNA library targeting >2000 cancer-relevant genes [57]. Among the 36 hits from this screen, 18 could be linked to NF-κB signaling. The top nine genes affecting NF-κB signaling (*RIPK1*, *c-FLIP*, *RELA*, *PRKCH*, *CCNB1*, *BCL2*, *NR4A2*, *TNFSF15*) were further investigated with independent siRNAs, and all were validated to increase sensitivity to erlotinib in H1650 cells as well as the TKI-sensitive cell lines HCC827 and H3255. NF-κB signaling plays a key role in inflammation and the innate immune response; however, its dysregulation has also been reported to promote angiogenesis and cell cycle progression and suppress apoptosis in multiple cancer types [68]. The hits were next evaluated in human bronchial epithelial cells (HBECs) that were modified to express either wild-type (WT) or mutant EGFR (L858R or ex19del). HBECs are normally resistant to erlotinib; however, individually silencing the screen hits induced erlotinib sensitivity in HBEC-EGFR^L858R^ and HBEC-EGFR^ex19del^, while no effect was observed in HBEC-EGFR^wt^ cells. To further validate the role of NF-κB in erlotinib resistance, they induced NF-κB by suppressing IκB, a negative regulator of NF-κB signaling, and found that this rescued cells from erlotinib-mediated toxicity. In patients, low IκB, indicative of active NF-κB signaling, is predictive of poor prognosis in patients treated with EGFR TKIs but is not associated with outcomes in patients treated with standard chemotherapy. This study highlights how NF-κB signaling allows EGFR mutant cells to overcome EGFR inhibition while also providing a rationale for targeting NF-κB in patients with mutant EGFR.

In a separate shRNA screen, CK1α was identified as a protein whose knockdown could prevent both intrinsic and acquired resistance to erlotinib. Lantermann and colleagues [53] transfected HCC827, HCC4006, and PC9 cells with ~6500 shRNAs targeting 350 cancer-relevant genes; they treated the cells with erlotinib or DMSO and collected and sequenced the cells after 10 and 24 days. shRNAs for *CSNK1A1*, encoding CK1α, were found to be depleted after erlotinib treatment in all three cell lines. Further functional validation reveals that the suppression of CK1α decreased erlotinib IC_50_ while also suppressing the amount of DTP cells that survive the initial pulse of the drug. Microarray analysis demonstrated significant upregulation of the NF-κB signaling pathway in DTP cells, which was inhibited with the expression of CK1α shRNAs. IKK negatively regulates IκB, itself a negative regulator of NF-κB [69]. The combination of erlotinib with AFN700, an IKK inhibitor, increased erlotinib sensitivity, whereas AFN700 alone had no effect on cells. Although this study used similar methodology and models as the work done by Bivona and colleagues [57], this data adds more supporting evidence to lung cancer cells expressing mutant EGFR being dependent on NF-κB signaling for survival; the inhibition by CK1α suppression or other NF-κB signaling components may have a therapeutic benefit in EGFR mutant NSCLC patients.

CK1α was also a hit in a third shRNA screen. Casás-Selves and colleagues [56] performed a whole-genome shRNA screen in HCC4006 and H322C cells treated with gefitinib. This study focused on the role of CK1α within the Wnt signaling pathway. CK1α was found to both activate and suppress Wnt signaling [70,71] and is known to play a role in multiple pathways linked to cancer development [72]. In this study, both the inhibition of CK1α by shRNA and the stimulation of CK1α by treatment with an activator increased erlotinib sensitivity [56]. Although CK1α may play an important role in allowing cells to bypass EGFR dependency, the mechanism remains poorly understood.

##### Urea Cycle Signaling

Metabolic dysregulation is a hallmark of cancer [73]. EGFR has been previously shown to induce metabolic reprograming through PI3K/AKT pathway activation [74] or c-Myc upregulation [75]; however, specific dependencies in NSCLC remain unclear. To elucidate potential metabolic vulnerabilities in the context of EGFR inhibition, Pham-Danis and colleagues [45] performed a synthetic lethal shRNA screen targeted to metabolic enzymes previously implicated in cancer. They utilized the EGFR mutant cell lines PC9, HCC4006, and H1650 as well as the EGFR^wt^ cell line H322C and PC9 cells that were made resistant to erlotinib through the expression of EGFR^T790M^. CSP1, a rate-limiting enzyme for the urea cycle, was found to be synthetically lethal with erlotinib in several EGFR mutant cell lines through this screen. Importantly, CSP1 inhibition also had additive effects when combined with osimertinib, a third-generation and more clinically relevant EGFR TKI. CSP1 inhibition also sensitized ELM4-ALK-driven cells to the ALK inhibitor crizotinib; however, it had no effect when combined with chemotherapy in various cells. This suggests that other tyrosine kinase-driven malignancies may also be dependent on the urea cycle for survival. Further functional investigation revealed that CSP1 knockdown combined with EGFR inhibition slows cell cycle progression as well as decreases glycolytic activity and the oxygen consumption rate. Overall, the combination further slows central carbon metabolism, dampens pyrimidine biosynthesis, and impairs arginine metabolism. In patients, CSP1 is highest in tumors with LKB1 loss and is correlated with poorer patient prognosis in stage I/II LUAD but not in later stages, suggesting a role for CSP1 in tumor initiation and/or progression. Overall, CSP1 and the urea cycle were found to play an important role in maintaining the survival of mutant EGFR cells treated with TKIs and represent a potential therapeutic vulnerability in this context.

##### FGFR

Another mechanism of resistance commonly seen in TKI-treated EGFR mutant NSCLC is a shift to a more mesenchymal phenotype that is less dependent on EGFR signaling [76]. The transcriptional induction of genes controlling epithelial to mesenchymal transition (EMT) has also been observed in cells entering a DTP state in response to EGFR TKI treatment [77]. Raoof and colleagues [46] performed a whole-genome CRISPR screen on two mesenchymal-like cell lines derived from patients that progressed on from EGFR TKIs. The top target for re-sensitizing cells to EGFR inhibition was FGFR1; FGFR1 and one of its ligands, FGF2, were also found to be associated with a more mesenchymal phenotype in EGFR mutant cell lines. FGFRs are a family of receptor tyrosine kinases that regulate cell migration, proliferation, growth, and survival [78]; they have also been previously reported to contribute to EGFR TKI resistance [79,80,81]. In mesenchymal-like drug-tolerant cells, the inhibition of EGFR and FGFR1 synergizes, whereas the combination shows no synergy in epithelial-like cells. The emergence of more mesenchymal DTP cells resulting from EGFR mutant cells treated with EGFR TKIs was found to be dependent on FGFR3. Both in vitro and in vivo combinations of EGFR and FGFR inhibitors in treatment-naïve cells suppressed the emergence of the DTP population and prevented acquired resistance. This study demonstrates that FGFR signaling is a key mechanism for cells to bypass EGFR dependence and indicates the potential efficacy of co-targeting EGFR and FGFR in patients with EGFR mutant NSCLC.

FGFR was also a hit in the genome-wide CRISPR screen performed by Zeng and colleagues [44], which detected YAP1 signaling as a positive moderator of EGFR signaling. This screen was also aimed at uncovering genes that contribute to the emergence of DTP cells, although performed on a different EGFR mutant cell line. Although the authors did not further investigate FGFR1, its detection in this screen further validates it as an important mediator of EGFR dependency.

##### Aurora Kinase A

Shah et al. [47] aimed to elucidate the genes driving residual disease that survives EGFR inhibition. They performed a drug screen on H1975 cells that were made resistant to rociletinib through long-term culture, aiming to uncover compounds that would synergize with the TKI and re-sensitize H1975 cells to EGFR inhibition. Two Aurora kinase inhibitors present in the screen, AZD1152 and VX680, were the top synergistic hits [47]. Aurora kinases are highly conserved kinases that play a key role in mitosis by regulating centrosome function, spindle assembly, chromosome alignment, and cytokinesis; however, they have also been found to be overexpressed in multiple cancers, suggesting a link to tumorigenesis [82]. Aurora kinase A (AURKA) was found to be overexpressed in the rociletinib-resistant H1975 cell line, and the addition of AURKA inhibitors was found to induce apoptosis through upregulation of BIM, re-sensitizing these cells to EGFR inhibition. The authors demonstrated that AURKA activity is induced by EGFR inhibition and is responsible for the initial establishment of drug-tolerant cell populations, with overexpression of AURKA, but not AURKB nor AURKC, in EGFR TKI-sensitive cells, resulting in drug resistance. AURKA inhibitors were also shown to be very effective in combination with EGFR inhibitors as first-line treatment in TKI-sensitive EGFR mutant cells. When evaluated on patient-derived xenograft models of EGFR mutant NSCLC, the AURKA inhibitor plus rociletinib or osimertinib was significantly better at inhibiting tumor growth. Finally, the authors also found a link between AURKA activity and TPX2 levels, an activator of AURKA signaling, suggesting that TPX2 may be used as a biomarker for AURKA-driven resistance to EGFR TKIs. This study highlights the role AURKA plays in the maintenance of drug-tolerant cells following EGFR inhibition. The combination of osimertinib and alisertib (MLN8237), the most clinically advanced AURKA inhibitor, is currently being evaluated in patients with Osimertinib-resistant LUAD (NCT04085315). Early results suggest an acceptable safety profile as well as clinically meaningful efficacy [83].

##### Sertraline

Jiang and colleagues [49] used a bioinformatics- and genetics-based approach to identify new potential indications for over 1000 FDA-approved drugs. They started by creating a comprehensive drug-gene interaction map using three public databases: DrugBank [84], PharmGKB [85], and Therapeutic Target Database [86]. In parallel, they made a global disease-gene association model from four different data sources: the OMIM [87], HuGE Navigator [88], PharmGKB [85], and Comparative Toxicogenomics databases [89]. From these datasets, they created a model to predict new indications for established drugs. Sertraline, an anti-depressant, was one of the top-rated hits. Sertraline and erlotinib synergize to inhibit proliferation in EGFR mutant and EGFR^wt^ NSCLC cell lines; however, these drugs have no synergy when tested in normal lung cells. Sertraline was found to suppress the AMPK/mTOR/S6K signaling axis. When combined with erlotinib, sertraline treatment results in increased autophagic flux, which was found to be the key contributor to the observed cytotoxicity. In an in vivo model of EGFR mutant NSCLC, sertraline enhanced the therapeutic efficiency of erlotinib. Sertraline has a favorable safety profile and good tolerability in patients and was found to be enriched 67-fold in lung tissue following postmortem analysis [90]. This suggests high concentrations of the drug can be achieved in the lungs, although the enriched sertraline in this instance may only reflect postmortem redistribution. Overall, the combination of sertraline and erlotinib offers a potential therapeutic strategy for NSCLC and highlights the role of AMPK/mTOR/S6K in the maintenance of EGFR mutant cells.

##### Bosutinib

Kim et al. [54] used a genetics and bioinformatics approach to uncover signaling dependencies in EGFR mutant lung cancer and repurpose existing drugs. They started by performing an shRNA screen in H1650 cells, a cell line bearing mutant EGFR that is insensitive to EGFR inhibitors. The screen consisted of ~3700 shRNAs targeting ~600 kinases and was aimed at determining what other kinases H1650 cells are dependent on for survival. Next, they performed RNA sequencing to determine which kinases were differentially expressed between H1650 cells and normal type II alveolar cells. By integrating the screen and transcriptional regulation data, they aimed to identify kinases that were important for both cancer cell transformation as well as survival. *CDK6*, *EGFR*, *MARK3*, *PBK*, *TBK1*, *DDR1,* and *EPHA4* were shared between the two approaches [54]. They next queried K-Map [54], a web-based program that connects kinases with drugs based on inhibitor IC_50_ and K_D_, to uncover drugs that inhibit the top essential and transformative kinases. Bosutinib, a Scr and Abl dual inhibitor, was among the highly ranked drugs. Bosutinib was found to have a lower IC_50_ than gefitinib or sorafenib in H1650 and H1975 cells, although the cells are still relatively resistant to bosutinib alone. The combination of gefitinib and bosutinib was found to be synergistic in the two cell lines studied and was found to increase apoptosis. No further mechanism for the action of bosutinib was provided.

##### GRB2

Chen and colleagues [43] utilized a similar approach as the one described above for bosutinib. They first analyzed a gene expression dataset consisting of EGFR TKI-sensitive and -resistant NSCLC cell lines in order to determine gene expression patterns associated with resistance. Next, they used Connectivity Map (CMap) [91], a computational tool that uses a reference database of gene expression signatures induced by specific drugs to define those most likely to “reverse” the gene profile associated with a particular disease state. Through CMap, they aimed to uncover unexplored drug-target connections relevant to NSCLC, which revealed that lymecycline, a semisynthetic derivative of tetracycline, may be useful in overcoming EGFR TKI resistance [43]. Further bioinformatics analyses revealed that GRB2 is the top target of lymecycline. GRB2 is an adaptor protein that binds EGFR and is essential for EGFR phosphorylation and regulation of downstream targets, including AKT and ERK pathways [92,93] (Figure 1A). In vitro, lymecycline inhibits mTOR, AKT, ERK, and STAT3 phosphorylation and induces apoptosis. Lymecycline alone can inhibit the growth of EGFR TKI-resistant cell lines. When combined with icotinib (a first-generation EGFR TKI) in an in vivo model of treatment-naïve EGFR-driven NSCLC, lymecycline slowed acquired resistance. This study highlights the importance of GRB2 as a mediator of canonical EGFR signaling reactivation. The combination of GRB2 and EGFR inhibition is effective in both EGFR TKI-resistant cells and in EGFR TKI-sensitive cells as first-line treatment.

##### Canonical MAPK and PI3K/AKT Signaling Reactivation

Open reading frame (ORF) or cDNA screens offer an alternative approach to determining the role of genetic changes detected in patients. In EGFR mutant NSCLC, this approach has been applied to determine how genetic changes can modulate sensitivity to EGFR inhibitors. Several ORF screens performed on EGFR mutant NSCLC lines have discovered novel mutations as well as previously characterized oncogenes that can help cells escape EGFR dependency through reactivation of the MAPK and PI3K/AKT pathway.

Sharifnia and colleagues [55] performed an ORF-based screen on EGFR mutant NSCLC cells treated with erlotinib. Their screen was focused on 589 ORFs coding for kinase or kinase-related genes, performed in the EGFR mutant cell line PC9; 18 ORFs rescued PC9 cells treated with erlotinib [55]. As PC9 cells are sensitive to EGFR inhibition, these ORFs encode genes that allow cells to bypass EGFR dependency. The 18 hits were evaluated in four additional EGFR mutant NSCLC lines, and, of those 18, only 6 (*CRKL*, *SRC*, *RAF1*, *FRK*, *BLK*, and *HCK*) universally rescued EGFR dependence, while the rest of the hits varied in effect across the cell line panel. A subset of the genes was also found to rescue ALK dependence in ELM4-ALK NSCLC cells, but no genes rescued cells from chemotherapy, suggesting these genes may help escape broader kinase dependence. Gene expression signatures associated with induction of these EGFR bypass genes were analyzed and found to be anti-correlated with signatures associated with MEK and PI3K inhibitors, suggesting that these genes may signal through these pathways. Indeed, co-inhibition of MEK and PI3K/mTOR restores sensitivity to EGFR inhibitors when *CRKL*, *SRC*, *RAF1*, *FRK*, *BLK*, and *HCK* are overexpressed in EGFR mutant cells. The sensitivity to MEK and PI3K inhibition highlights the important role of ERK/MAPK and AKT signaling reactivation following EGFR inhibition as a way of bypassing the need for mutant EGFR signaling.

Bolan and colleagues [42] performed a separate genome-wide ORF overexpression screen in PC9 cells, aimed to calculate the fitness of specific somatic mutations in response to challenge by first- and third-generation EGFR inhibitors as well as MEK inhibitors (MEKi). For each treatment, different ORFs were clustered in tiers based on the resistance they conferred. ORFs encoding genes that signal through the MAPK and PI3K pathways were enriched among the resistant genotypes, highlighting the important role reactivation that these two pathways can play in driving EGFR TKI resistance. The addition of MEKi was sufficient to re-sensitize most mutant ORFs to EGFR inhibition. Twenty-three of the genotypes investigated resulted in resistance to the combination of EGFR and MEK inhibitors. This group was positively enriched with genotypes that positively regulate the MAPK pathway, including KRAS^G13D^, BRAF^V600E^, and EGFR^T709M/L858R/C797S^, again highlighting the key role that MAPK signal reactivation plays in driving resistance.

#### 2.1.2. Negative Moderators of EGFR Dependency

##### KEAP1

Several groups have identified KEAP1 as a key negative regulator of EGFR dependency in mutant cells. In a genome-wide CRISPR screen performed on multiple NSCLC cell lines bearing different driver mutations and treated with different targeted therapies, Krall and colleagues [50] identified KEAP1 loss as a key regulator of resistance to multiple targeted therapies, including erlotinib in EGFR mutant NSCLC cells. KEAP1 negatively regulates NRF2 by targeting it for proteasomal degradation [94]. To determine whether NRF2 activity is driving resistance to EGFR inhibition, researchers overexpressed mutant forms of NRF2 and found these were sufficient to promote resistance to erlotinib. In KEAP1-deficient cell lines, re-expression of KEAP1 increases sensitivity to targeted therapies. As KEAP1 and NRF2 are known to respond to oxidative and electrophilic stress, the authors investigated if this function was involved in drug resistance. Treatment with erlotinib and other MAPK pathway inhibitors was found to induce reactive oxygen species (ROS), and *KEAP1* knockout was found to rescue cells from drug-induced ROS through the increase of glutathione synthesis. *KEAP1* knockout was also found to induce alterations to cellular metabolism, allowing cells to proliferate in the absence of MAPK signaling.

*KEAP1* was also detected as a hit in two other genome-wide CRISPR-Cas9 screens performed by Zeng and colleagues [44] and Terai and colleagues [48]. Although KEAP1 was not investigated further in either study, its detection in these studies further reinforces its importance as a mediator of EGFR dependence.

##### ARIH2

In the genome-wide CRISPR-Cas9 screen performed by Zeng et al. [44], guides targeting *YAP1*, *RIC8A,* and *LPAR2* were found to be significantly depleted in the cells that survived erlotinib, indicating that the encoded proteins aid cellular escape from EGFR dependence. Among the significantly enriched guide RNAs were known tumor suppressors *KEAP1* and *FBXW7* as well as several members of the Cullin 5 (CUL5)-RING E3 ligase (CRL5) complex, a previously undescribed mechanism of resistance [44]. The CRL5 complex targets proteins for proteasomal degradation, with many of its substrates constituting known oncogenes or tumor suppressors [95]. *ARIH2*, a member of the CRL5 complex, was further investigated as the top driver of resistance to erlotinib. *ARIH2* knockout results in an increased fraction of cells that enter a DTP state following treatment with an EGFR inhibitor and results in increased tumor size in vivo. Mechanistically, *ARIH2* or *CUL5* knockout were found to result in increased protein levels of METAP2, ALDOA, and PSAT1 despite no changes to transcript levels, suggesting post-transcriptional regulation. METAP2 regulates global protein synthesis and co-translationally removes N-terminal methionine from nascent proteins, while PSAT1 and ALDOA are important enzymes involved in serine biosynthesis and glycolysis pathways, respectively. Overexpression of those genes was found to promote EGFR TKI resistance. As *ARIH2* and other members of the CRL5 complex are involved in proteasomal degradation by ubiquitination, the authors hypothesized that *ARIH2* targets METAP2, ALDOA, and PSAT1 for ubiquitination, although they were unable to demonstrate a direct link. Overall, the loss of *ARIH2* or other members of the CRL5 complex can result in resistance to EGFR inhibition through the post-transcriptional modulation of multiple proteins, although specific mechanisms need to be systematically investigated in the future.

##### Ufmylation Pathway

In another study aimed at uncovering the emergence of DTP populations following EGFR TKI treatment, Terai and colleagues [48] performed a genome-wide CRISPR-Cas9 screen in EGFR-dependent NSCLC cells treated with erlotinib and THZ1. DTP cells survive by undergoing epigenetic changes and transcriptional adaptations that promote cell survival, which can be partially reversed by combining erlotinib with THZ1, a compound that was found to inhibit RNA-polymerase-2-dependent transcription. Among the top hits of the genes that suppressed the synergy observed when combining erlotinib and THZ1 were multiple components of the ufmylation pathway (*UFM1*, *UFSP2*, *UBA5*, *UFC1*, and *UFL1*), a recently described post-transcriptional modification pathway [48]. Ufmylation has been demonstrated to regulate proteostasis, a network responsible for protein folding, maintaining conformational stability, and degrading unfolded proteins [96]. Loss of ufmylation does not trigger the activation of canonical EGFR signaling pathways. Instead, they found that it triggers a protective unfolded protein response associated with the upregulation of STING. In DTP cells, the induction of STING is also associated with activation of NF-κB signaling as well as unfolded protein response (UPR) gene activation. Although ER stress can induce cell death, there is growing evidence that tolerable levels of ER stress may be pro-tumorigenic and immunosuppressive [97,98]. Loss of ufmylation and the subsequent increase in ER stress signaling was also accompanied by a dependence on Bcl-x_L_, suggesting a potential weakness in EGFR TKI resistance cases where the ufmylation pathway is inactivated.

##### Capicua

Liao and colleagues [51] performed both CRISPR-Cas9 and shRNA screens with RNAs targeting ~500 genes for which loss of function had previously been reported to play a role in tumorigenesis. The aim was to uncover the modifiers of EGFR sensitivity in PC9 cells treated with gefitinib. *CIC* was a hit in both the CRISPR-Cas9 and shRNA branches of the study [51]. *CIC* loss also conferred resistance to erlotinib and osimertinib in PC9 as well as osimertinib resistance in H1975 cells, a cell line insensitive to erlotinib due to their T790M mutation. While the loss of *CIC* did not protect cells from apoptosis, it did prevent the cell cycle arrest normally induced by gefitinib. Treatment with palbociclib, a CDK4/6 inhibitor, restored some sensitivity to gefininib, while palbociclib alone had no effect on cells. Gene set enrichment analysis on transcripts differentially regulated in *CIC*-deficient cells determined by RNA-seq revealed that EGFR and ERK1/2 target genes were upregulated. *CIC* loss had no effect on either phospho-EGFR or phospho-ERK1/2, suggesting that CIC regulates genes downstream of these effectors. Of the genes found to be upregulated following *CIC* knockdown, *ETV1* conferred a significant growth advantage in the presence of gefitinib. Overall loss of *CIC* was found to promote cell survival upon EGFR TKI treatment by upregulating EGFR and MAPK target genes as well as promoting cell cycle entry. CIC mutations have also been detected in separate models of acquired resistance to osimertinib [99], further emphasizing its importance in EGFR signaling.

##### SWI/SNF

In the same screen that identified *CIC* loss as driving EGFR TKI resistance, multiple components of the SWI/SNF complex, notably *PBRM1*, *ARID2*, and *ARID1A*, were also identified as hits [51]. The role of PRMB1 in cancer remains unclear, and while no direct links between *PRMB1* and EGFR signaling have been established, it has previously been linked to p21 expression and cell cycle arrest in breast cancer [100]. *PRMB1* loss was found to have no effect on p21 after gefitinib treatment or any effect on EGFR phosphorylation or expression levels. The authors did observe that *PRMB1*-knockout cells had a slower reduction of phospho-AKT following EGFR inhibition, suggesting *PRMB1* may attenuate the effects of EGFR signaling suppression by activating AKT signaling. The mechanistic role of *ARID2* and *ARID1A* loss in the context of gefitinib resistance is still being investigated.

##### miRNA

MicroRNAs (miRNA) are involved in almost every facet of cellular function and are dysregulated in different cancers. To identify miRNAs that might play a role in EGFR inhibitor resistance, Pal and colleagues [41] performed a miRNA overexpression screen with ~2000 human-encoded miRNAs in EKVX and H322M cells treated with erlotinib. miR-5693, miR-3618, and miR-432-5p were reported to promote resistance to erlotinib, gefitinib, and afatinib in EGFR mutant NSCLC cells [41]. Drug efflux was the pathway predicted to be most affected by the hits from this screen, followed by PI3K/AKT signaling, although no mechanistic validation of the effects was performed in this study. miR-432 was found to be overexpressed in NSCLC tumor samples. This study highlights the potential role miRNAs can play in resistance, although overexpressed miRNAs have yet to be discovered as inducing resistance to EGFR TKIs in patients.

### 2.2. Cooperating Genomic Alterations in EGFR Mutant NSCLC Cells

**Figure 2:**
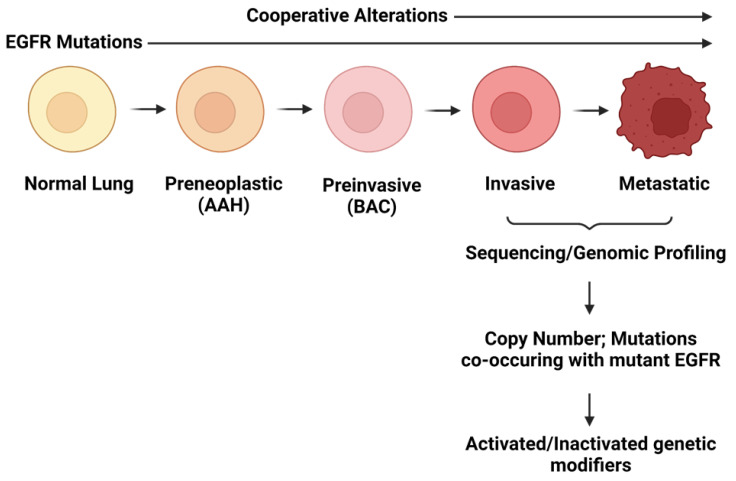
EGFR mutations occur early in lung tumorigenesis but are insufficient to cause invasive tumors. Co-operative alterations are gained through the stepwise progression of EGFR mutant lung cancer cells, which include genetic and epigenetic changes that activate/inactivate genes modifying the tumorigenic capacity of the cells. These alterations aid the transition of cells to invasive adenocarcinoma. Figure made with BioRender.

While our understanding of how mutant EGFR drives lung tumorigenesis has greatly advanced, many key questions have yet to be resolved. Perhaps the most outstanding issue is determining patterns of gene disruption that are selected for during tumorigenesis. This is an important consideration as experimental evidence suggests that multiple genetic alterations are required to transform normal lung cells and drive them to full malignancy [8]. For example, model systems have revealed that mutant EGFR alone is not sufficient for tumorigenesis [7,9]. This is exemplified by transgenic mouse models expressing mutant EGFR in the lung epithelium, where the variable latency period between transgene induction and the onset of lung tumors implies that secondary alterations are a requirement for full malignancy [101]. Furthermore, mutant EGFR has been detected in histologically normal lung epithelium in patients, and immortalized lung epithelial cell lines transduced with mutant EGFR have failed to progress to a fully malignant phenotype [7,9]. Therefore, although tumors expressing this mutant oncogene are clearly dependent on its sustained expression for survival, these findings suggest that additional genetic/epigenetic alterations cooperate with mutant EGFR, activating/disrupting genes that “modify” tumorigenic capacity in lung cancer development (Figure 2). Identifying these genetic modifiers of oncogene-induced tumorigenesis is imperative to determining the mechanisms of tumor progression and, subsequently, identifying new targets for anticancer agents that improve patient outcomes. These modifiers may represent logical targets to design combination-based therapies that counteract the inevitable drug resistance and tumor recurrence that occurs with single-agent TKIs. Here, we detail efforts made to comprehensively identify and catalog genes that are disrupted in EGFR mutant patient lung tumors and describe potential candidates that may provide new targets for therapeutic intervention.

#### 2.2.1. Copy Number Alterations

EGFR mutant NSCLC genomes display numerous regions of copy number gains and losses. While some of these are found at similar frequencies in other molecular subsets of LUAD, others are more specific to mutant EGFR tumors. Below, we detail the most significant copy number changes associated with EGFR mutant NSCLCs and highlight the potential candidate genes they affect (summarized in Table 2).

##### Loss of Chromosome arm 8p Encompassing *DUSP4* and *DOK2*

One of the most notable copy number alterations associated with EGFR mutant LUAD is chromosome arm 8p loss [110]. While the minimal region of this loss contains numerous genes, two have specifically been linked to mutant-EGFR-driven tumorigenesis. *DUSP4* is a putative tumor suppressor that is located at 8p12, which frequently undergoes a single-copy genomic loss in EGFR mutant LUADs [109,110]. Also located on chromosome arm 8p, *DOK2* undergoes single copy loss alongside *DUSP4* in EGFR mutant LUADs. *DOK2* was demonstrated to be a tumor suppressor, where its overexpression inhibited the tumor-forming ability of EGFR mutant LUAD cells and its loss led to oncogenic EGFR-driven tumorigenesis in vivo [113]. Furthermore, the co-deletion of *DUSP4* and *DOK2* is haploinsufficient and results in the synergistic activation of MAPK signaling to promote cell proliferation [111]. In particular, *DUSP4* loss may cooperate with EGFR mutation to disrupt the negative feedback control of MAPK signaling and fully enable its activation [110]. The effects of concomitant *DOK2* and *DUSP4* loss in mice were verified to be significantly associated with poor survival outcomes in clinical data [111].

##### Amplification of Chromosome Arm 7p: *EGFR* and *LANCL2*

Mutant-specific allele imbalance associated with copy number gains is typical of many oncogenes, including *EGFR* in NSCLC [112]. EGFR lies on chromosome 7, which is typically gained and/or amplified in NSCLC tumors [119]. Amplification of the mutant *EGFR* allele alters EGFR protein levels and increases downstream signaling activity, which has been demonstrated to function as a tumor progression event [109,112]. As part of the alteration encompassing *EGFR*, *LANCL2* is also amplified [119]. A recent study found a connection between the role of LANCL2 in AKT hyperactivity and the role of AKT hyperactivity in promoting EGFR mutant LUAD cell proliferation [114]. This study also identified two novel protein interactors of LANCL2, filamin A (FLNA) and glutathione S-transferase Mu 3 (GSTM3), which may be involved in EGFR mutant LUAD tumorigenesis [114].

##### Chromosome 16p: *GGA2*

We have recently demonstrated through a comprehensive comparison of EGFR mutant and wild-type LUADs that along with chromosome 7p gain and 8p loss, 16p gain is specific to EGFR mutant tumors. Through integrative genomic analyses, we pinpointed *GGA2* as a target of this amplified region [109]. As a clathrin adapter protein, GGA2 is involved in protein-sorting trafficking and has been shown to help stabilize activated EGFR to increase EGFR-driven tumorigenesis [109]. Thus, much like the amplification of EGFR itself, GGA2 may work through increasing active mutant EGFR protein levels in lung cancer cells to promote growth.

##### *NKX2-1* 

Multiple studies have recently demonstrated *NKX2-1* amplification in EGFR mutant LUAD [102,103,115]. As a master transcription factor, Homeobox protein Nkx-2.1 mediates its effect through transcriptional regulation of downstream targets. Through a combined transcriptome and cistrome analysis, EGFR was identified as a downstream target of interest, although the exact mechanism remains unknown [116].

##### *MDM2* 

*MDM2* amplification is observed in 12% of EGFR mutant LUADs. The MDM2 protein regulates proteosome-dependent p53 degradation and can also directly remove p53 from cells. Its amplification and likely subsequent loss of the tumor suppressor, p53, may be involved in its tyrosine kinase inhibitor resistance mechanism [117,120].

##### Cyclins and Cyclin-Dependent Kinases

In a normal cell, the entry of G1/S is controlled through RB1 phosphorylation by the CCND1-CDK4/6 complex to activate E2F transcription factors [121]. CDK4 is amplified in 10% of EGFR mutant LUADs [102]. Other genes responsible for G1/S entry control are also amplified in baseline EGFR mutant patient samples, such as *CDK6* (7%) and *CCNE1* (6.9%) [105]. Notably, a correlation exists between *CDK4/6* amplification and first-generation EGFR TKI resistance, which has led to ongoing studies of EGFR and CDK4/6 inhibitors [122].

#### 2.2.2. Mutations

##### TP53

Tumor protein p53 is frequently found to contain a missense mutation in patients harboring EGFR mutations [102,103]. Similar results are observed in The Cancer Genome Atlas cohort. Recently, Zheng et al. found that LUAD patients with coexisting EGFR and TP53 mutations have a poorer prognosis, likely through the upregulation of COMP and ITGB8 [104].

##### *RB1* 

As a tumor suppressor gene, *RB1* is mutationally inactivated in around 10% of EGFR mutant LUADs [102,103,105]. Most of these *RB1* mutations tend to co-occur with *TP53* mutations, where both genes are highly involved in cell cycle control [118]. Furthermore, tumors with inactivation of both *TP53* and *RB1* have been shown to be associated with small cell lineage transformation and TKI resistance in EGFR mutant lung cancer [123].

##### *PIK3CA* 

*PIK3CA* mutations are seen in 12% of EGFR mutant LUADs, a proportion that includes both classical kinase and helical domain mutations [102,106]. The activating mutation results in constitutive AKT-mTOR pathway activation, which leads to tumor survival and proliferation [124].

##### *CTNNB1* 

β-catenin, encoded by *CTNNB1*, is co-mutated alongside *EGFR* in EGFR mutant LUADs across several studies [102,105,107,108]. The protein translocates into the nucleus in EGFR mutant LUAD cells as a nuclear transcriptional activator and engages in WNT signaling [125].

##### *NF1* 

The *NF1* gene is a tumor suppressor that fails to downregulate Ras signaling when mutated [126]. The gene encodes a RAS GTPase activating protein that is inactivated by a deleterious mutation in around 9.4% of EGFR mutant patients. While the gene mutation does tend to co-occur with EGFR mutation, it is more studied as a mechanism of TKI resistance [127].

## 3. Conclusions

EGFR mutant NSCLC has been one of the main templates for oncogene addiction since the concept was first proposed [128]. Pre-clinical models of EGFR mutant NSCLC tended to be highly sensitive to EGFR inhibitors, while these same inhibitors garnished strong initial responses from patients in the clinic. However, despite the undeniable improvements that EGFR TKIs present over chemotherapy for patients bearing mutant EGFR, a decade and a half of targeting mutant EGFR has still not yielded the long-term improvement in patient survival initially hoped for. EGFR mutant NSCLC now reflects an amended view of oncogene addiction [129], one that can be escaped following the selective pressure applied by EGFR inhibitors due to heterogeneity within the tumor as well as epigenetic and genetic adaptations. Profiling of tumors with acquired EGFR TKI resistance has uncovered many such changes across populations of patients. Additionally, within individual patients, there likely exists multiple subpopulations that are resistant to EGFR TKIs, making treatment by sequential monotherapies an unattractive solution to target drug-resistant tumors, even before considering the challenges of appropriately dosing each drug.

**Figure 3:**
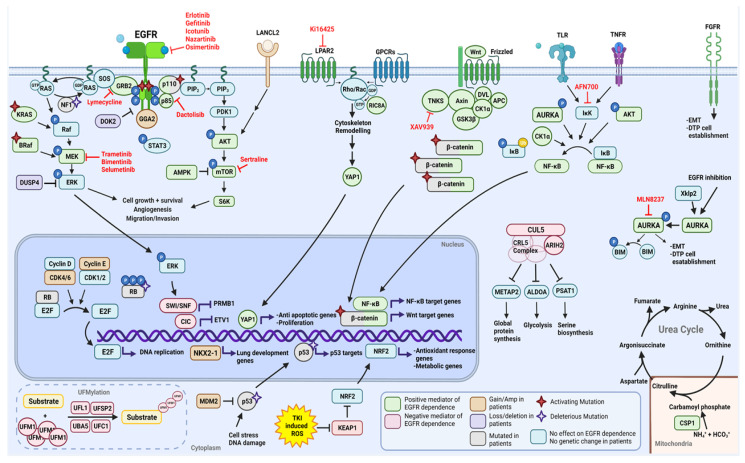
Proteins, signaling pathways, and drugs discovered to modulate sensitivity to EGFR inhibitors, as well as proteins found mutated, amplified, or lost in patient samples prior to treatment with EGFR inhibitors. Figure made with BioRender.

Targeting factors known to drive EGFR dependence before the emergence of distinct drug-resistant subpopulation profiles is a way of improving EGFR TKI effectiveness in first-line treatment while also potentially limiting the challenges of treating drug-resistant tumors with unknown mechanisms of resistance. Here, we present a summary of the results of high throughput genetic, drug, miRNA, and profile screens performed on models of EGFR mutant NSCLC aimed at determining factors that positively or negatively affect mutant EGFR signaling dependence and a summary of the genetic changes that commonly co-occur with mutant EGFR in TKI-naïve patients (Figure 3).

The overlap between these two groups highlights pathways that may be of key importance to overcoming EGFR TKI resistance. Co-occurring alterations such as *DUSP4* and *DOK2* loss, NF1 deleterious mutation, and *GGA2* gain all promote signaling downstream of EGFR, while functional screen data has revealed lymecycline as a key sensitizer to EGFR inhibition by inhibiting GRB2 and shutting down EGFR signaling through ERK/AKT/STAT3. β-catenin mutations also co-occur with EGFR mutations and serve as a mediator of EGFR dependence. Activation of NF-κB emerges from several screens as a key mediator of EGFR dependence due to the myriad routes its signaling can be activated. Promising pre-clinical data with IKK and AURKA suggests a combination of EGFR and NF-κB signaling inhibition may benefit patients; this is currently being evaluated in patients (NCT04085315). FGFR1 signaling emerges as a key mediator for cells that escape EGFR inhibition by shifting to a more mesenchymal phenotype. With inhibitors already clinically available, this combination may soon serve subsets of patients. MET signaling was discovered through separate studies as a mediator of intrinsic resistance to EGFR TKIs. The combination of MET inhibitors and osimertinib is currently being trialed in patients (NCT02143466) and has yielded positive early results [130].

In the future, oncogenic drivers of tumorigenesis and mediators of EGFR TKI resistance may also be targeted by gene therapy. Adeno-associated virus 9 (AAV9) vectors are currently the leading platform for gene therapy delivery [131] and can function as vectors for constructs directed at gene replacement to compensate for loss of function mutations or gene silencing through RNAi or CRISPR/Cas9. Gene therapy through AAV9 offers potential advantages for the treatment of cancer; shRNAs, so frequently in pre-clinical models, could be delivered to specific cell or tissue types in patients through specially designed capsids, bypassing the expensive and inefficient process of drug development, while also allowing the targeting of proteins for which no specific inhibitors have been developed. Indeed, many of the mechanisms of resistance to EGFR TKI inhibition—as well as mutant EGFR itself—are potential targets for AAV9 gene therapy. However, to date, only two AAV9-based therapies have been FDA approved, neither pertaining to cancer. Identification of lung-cancer-specific extracellular markers for cancer-specific delivery of genetic material without affecting normal cells or, alternatively, the design of shRNAs targeted specifically to mRNA coding for oncogenic driver mutations will be necessary for gene therapy to be plausible. This is of added importance in lung cancer as many of the key oncogenic drivers have important functions in normal cells throughout the body. However, while promising, this technology still needs to be refined before it can be considered for use in lung cancer treatment.

Negative mediators of EGFR dependence are of equal importance to consider when planning therapeutic interventions. NRF2 is the master regulator of the cellular antioxidant response, and disruptions in the KEAP1-NRF2 signaling axis were found to provide a growth advantage to lung cancer cells as well as resistance to chemotherapy through control of a broad range of cellular functions, including redox homeostasis, metabolism, survival, and proliferation [132,133]. Along with chemoresistance, inactivation of KEAP1 and the subsequent activation of NRF2 and downstream pathways are also associated with decreased dependency on EGFR signaling in multiple functional screens. Genetic inhibition of NRF2 restores sensitivity to EGFR inhibition [50]; however, efforts to pharmacologically inhibit NRF2 are yet to yield any safe and specific candidates, in large part due to the similarity between NRF2 and other basic leucine zipper (bZIP) proteins. Additionally, NRF2 is important for oxidative stress response in normal cells, suggesting that its inhibition may elicit potential side effects. As a tumor suppressor frequently lost or mutated in NSCLC, KEAP1 profiles as a possible target for AAV9-mediated gene replacement therapy once the technology has been refined. Furthermore, targeting elements downstream of NRF2 may present an alternative and has shown some promise in pre-clinical models [134,135]. One study found that LKB1 or KEAP1 loss of function mutations were associated with prolonged overall survival in patients receiving anti-PDL1 immune checkpoint inhibitors, although this has yet to be observed in a cohort with EGFR mutant patients [136]. Other negative mediators of EGFR signaling are not sufficiently characterized at the current stage to inform clinical decisions. For example, the role of CRL5 and SWI/SNF biology in the context of EGFR TKI resistance is not fully understood, both having only recently been linked to resistance [44,51]. Likewise, ufmylation as a process has only been preliminarily described; however, cells resistant to EGFR TKI through ufmylation loss were found to be sensitive to Bcl-xL inhibition, which could be an avenue for therapeutic exploitation [48]. DTP cells emerging after challenge with EGFR TKIs have also been reported to be dependent on the suppression of apoptosis [137], and the non-selective BCL-2/Bcl-xL inhibitor ABT-263 is being trialed in combination with osimertinib in EGFR TKI-resistant NSCLC patients (NCT02520778). CIC loss has been reported to drive EGFR TKI resistance in specific models [99] and is associated with the upregulation of ETS family transcription factors, although this remains poorly understood as the suppression of these transcription factors only partially restores EGFR inhibitor sensitivity. Lastly, the co-administration of a CDK4-6 inhibitor was found to partially restore EGFR TKI sensitivity, specifically when CIC was lost [51]. Thus, despite promising biological roles in regulating EGFR dependency, negative regulators will require additional characterization before strategies for therapeutic development can be defined.

A necessary development needed for effectively targeting positive mediators of EGFR dependence will be the identification of biomarkers. For example, high levels of Xklp2 are associated with poorer prognosis in patients treated with EGFR inhibitors and are also associated with activation of AURKA, a hit from a drug screen. Patients with mutant EGFR and high levels of Xklp2 may benefit from AURKA inhibitors in conjunction with EGFR inhibitors, while patients with mutated PI3K may benefit from a combination of EGFR and PI3K inhibitors. Together, these studies highlight the broad range of pathways that affect EGFR signaling in NSCLC tumor cells. While work is still necessary to characterize the appropriate setting for combination therapy, targeting EGFR and its signaling dependencies offers a promising way to improve patient outcomes.

## Figures and Tables

**Table 1:** Pathways and drugs discovered to modulate sensitivity to EGFR inhibition through high-throughput functional and profiling screens performed on NSCLC cell lines.

Hits	Screening Methodology	Model	Drug Used	Year	Reference
**miR-5693, miR-3618, and m+B5:G22iR-432-5p**	2019 mature microRNAs	EKVX, H322M	Erlotinib	2021	[41]
**MAPK and AKT pathway**	17,255 ORFs, covering 12,728 genes and 35 mutant oncogenes	PC9	Erlotinib, Osimertinib, Trametinib	2020	[42]
**GRB2**	CMap analysis	PC9, HCC827	Icotunib	2020	[43]
**RIC8A, LPAR2, YAP1, ARIH2, KEAP1**	Whole genome CRISPR Cas9 screen (18,360 genes)	HCC827	Erlotinib	2019	[44]
**CPS1**	shRNAs targeting rate-limiting metabolic enzymes	PC9, HCC4006, H1650, H322C, PC9, PC9-EGFR T790M	Erlotinib	2019	[45]
**FGFR**	Whole genome CRISPR Cas9 screen (20,000 genes)	Patient Derived Cells	Nazartinib	2019	[46]
**Aurora kinase A**	94-compound cancer-focused drug library	H1975	Rociletinib	2019	[47]
**Ufmylation pathway**	Whole genome CRISPR Cas9 screen (18,454 genes)	PC9	Erlotinib, THZ1	2018	[48]
**Sertraline**	Comprehensive drug-gene interactions profile	H522, A549, H1975, PC9	Erlotinib	2018	[49]
**KEAP1**	CRISPR-Cas9 KO targeted screens	HCC827	Erlotinib	2017	[50]
**CIC, SWI/SNF**	CRISPR and shRNA library (10 gRNAs or shRNAs per gene) targeting 500 potential tumor suppressors	PC9	Gefitinib	2017	[51]
**YAP1**	shRNA screen (~60,000 individual shRNAs)	PC9	Cisplatin	2016	[52]
**CK1α**	shRNA screen against about 350 potentially cancer-relevant genes (~6500 shRNAs)	HCC827, HCC4006 and PC9	Erlotinib	2015	[53]
**Bosutinib**	3700 shRNAs targeting ~600 kinases	H1650	Gefitinib	2014	[54]
**CRKL, SRC, RAF1, FRK, BLK, and HCK**	589 ORFs encoding kinases and kinase related proteins	PC9	Erlotinib	2014	[55]
**Wnt/β-catenin pathway, tankyrase**	SBI shRNA library	H322C, HCC4006	Gefitinib	2013	[56]
**NF-κB**	shRNA screen targeting >2000 cancer-relevant genes	H1650	Erlotinib	2011	[57]

**Table 2:** Mutations commonly detected in parallel to mutant EGFR in patients prior to treatment.

Gene Name	Symbol	Chromosome	Alteration Type	Frequency	Pathway	Reference
**Tumour protein p53**	*TP53*	17p	Deleterious mutation	51–60%	P53	[102,103,104]
**RB transcriptional corepressor 1**	*RB1*	13q	Deleterious mutation	10–12%	RB/E2F (G1/S)	[102,103,105]
**Neurofibromatosis type 1**	*NF1*	17q	Deleterious mutation	9.40%	Ras	[105]
**Phosphatidylinositol-4,5-biphosphate 3-kinase catalytic subunit alpha**	*PIK3CA*	3q	Activating mutation	12%	PI3K-AKT	[102,106]
**Beta catenin 1**	*CTNNB1*	3p	Activating mutation	9%	WNT/β-catenin	[102,105,107,108]
**Golgi associated, gamma adaptin ear containing, ARF binding protein 2**	*GGA2*	16p	Gain/Amp	59%	EGFR	[109]
**Dual specificity phosphatase 4**	*DUSP4*	8p	Loss/Deletion	49% *	MAPK/ERK	[110,111]
**Epidermal growth factor receptor**	*EGFR*	7p	Gain/Amp	59%	EGFR	[112]
**Docking protein 2**	*DOK2*	8p	Loss/Deletion	48–63%	MAPK/ERK	[111,113]
**LanC like 2**	*LANCL2*	7p	Gain/Amp	37% **	Akt phosphorylation	[114]
**NK2 Homeobox 1**	*NKX2-1*	14q	Gain/Amp	15%	Regulates P53 transcription	[102,103,115,116]
**Mouse Double Minute 2**	*MDM2*	12q	Gain/Amp	12%	MDM2-p53	[102,117]
**Cyclin dependent kinase 4**	*CDK4*	12q	Gain/Amp	10%	CDK4/6 (G1/S)	[102,105,118]
**Cyclin dependent kinase 6**	*CDK6*	7q	Gain/Amp	7%	G1/S	[105,118]
**Cyclin E1**	*CCNE1*	19q	Gain/Amp	6.90%	G1/S	[105]
